# Association between different GLP-1 receptor agonists and gastrointestinal adverse reactions: A real-world disproportionality study based on FDA adverse event reporting system database

**DOI:** 10.3389/fendo.2022.1043789

**Published:** 2022-12-07

**Authors:** Lulu Liu, Jia Chen, Lei Wang, Chen Chen, Li Chen

**Affiliations:** ^1^ Department of Pharmacy and Evidence-Based Pharmacy Center, West China Second University Hospital, Sichuan University, Chengdu, China; ^2^ School of Pharmaceutical Science and Technology, Tianjin University, Tianjin, China; ^3^ Department of Pharmacy, Sichuan Provincial People’s Hospital Jinniu Hospital, Chengdu, China; ^4^ West China School of Pharmacy, Sichuan University, Chengdu, China; ^5^ Key Laboratory of Birth Defects and Related Diseases of Women and Children, Ministry of Education, Chengdu, China

**Keywords:** GLP-1 receptor agonists, gastrointestinal toxicities, adverse drug reactions, pharmacovigilance, data mining

## Abstract

**Objective:**

Glucagon-like peptide-1 receptor agonists (GLP-1 RAs) have significantly improved clinical effects on glycemic control. However, real-world data concerning the difference in gastrointestinal adverse events (AEs) among different GLP-1 RAs are still lacking. Our study aimed to characterize and compare gastrointestinal AEs among different marketed GLP-1 RAs (exenatide, liraglutide, dulaglutide, lixisenatide, and semaglutide) based on real-world data.

**Methods:**

Disproportionality analysis was used to evaluate the association between GLP-1 RAs and gastrointestinal adverse events. Data were extracted from the US FDA Adverse Event Reporting System (FAERS) database between January 2018 and September 2022. Clinical characteristics, the time-to-onset, and the severe proportion of GLP-1 RAs-associated gastrointestinal AEs were further analyzed.

**Results:**

A total of 21,281 reports of gastrointestinal toxicity were analyzed out of 81,752 adverse event reports, and the median age of the included patients was 62 (interquartile range [IQR] 54–70) years old. Overall GLP-1 RAs were associated with increased risk of gastrointestinal system disorders (ROR, 1.46; 95% CI, 1.44–1.49), which were further attributed to liraglutide (ROR, 2.39; 95% CI, 2.28–2.51), dulaglutide (ROR, 1.39; 95% CI, 1.36-1.42), and semaglutide (ROR, 3.00; 95% CI, 2.89–3.11). Adverse events uncovered in the labels included gastroesophageal reflux disease, gastritis, bezoar, breath odor, intra-abdominal hematoma, etc. Furthermore, it was observed that semaglutide had the greatest risk of nausea (ROR, 7.41; 95% CI, 7.10–7.74), diarrhea (ROR, 3.55; 95% CI, 3.35–3.77), vomiting (ROR, 6.67; 95% CI, 6.32–7.05), and constipation (ROR, 6.17; 95% CI, 5.72–6.66); liraglutide had the greatest risk of abdominal pain upper (ROR, 4.63; 95% CI, 4.12–5.21) and pancreatitis (ROR, 32.67; 95% CI, 29.44–36.25). Most gastrointestinal AEs tended to occur within one month. Liraglutide had the highest severe rate of gastrointestinal AEs (23.31%), while dulaglutide had the lowest, with a severe rate of 12.29%.

**Conclusion:**

GLP-1 RA were significantly associated with gastrointestinal AEs, and the association was further attributed to liraglutide, dulaglutide, and semaglutide. In addition, semaglutide had the greatest risk of nausea, diarrhea, vomiting, constipation, and pancreatitis, while liraglutide had the greatest risk of upper abdominal pain. Our study provided valuable evidence for selecting appropriate GLP-1 RAs to avoid the occurrence of GLP-1 RA-induced gastrointestinal AEs.

## Introduction

According to the reports of the International Diabetes Federation, there were 537 million patients with diabetes worldwide in 2021, and the prevalence of diabetes was estimated at 10.5% (1). Glucagon-like peptide-1 receptor agonists (GLP-1 RAs) have been increasingly recommended in the treatment of type 2 diabetes (T2DM) due to their favorable effects on improving glycemic control and reducing the risk of cardiovascular events (2, 3). GLP-1RAs lower the blood glucose of patients with diabetes through transient glucose-dependent stimulation of insulin, and suppression of glucagon secretion and gastric emptying (4, 5). So far, a total of seven GLP-1 RAs, including exenatide, liraglutide, albiglutide, dulaglutide, lixisenatide, semaglutide, and tirzepatide, have been approved by the US Food and Drug Administration (FDA) for treatment of T2DM.

With the extensive use of GLP-1 RAs, adverse drug reaction (ADR) reports are increasing gradually, which has attracted the attention of clinicians and drug administrations. The most frequently reported adverse events (AEs) of GLP-1 RAs were gastrointestinal adverse drug reactions, including nausea, vomiting, and abdominal pain, which were closely related to activation of central and peripheral GLP-1 receptors (6, 7).

In a recent network meta-analysis, overall GLP-1 RAs significantly increased the incidence of gastrointestinal AEs compared with placebo or conventional treatment, and the odds ratios for nausea, vomiting and diarrhea from GLP-1 RAs were 11.8 (95% CI, 2.89–46.9), 51.7 (95% CI, 7.07–415), and 4.93 (95% CI, 1.75–14.7), respectively (8). Other meta-analyses have also reported similar results (6, 9–11). Moreover, some gastrointestinal adverse reactions with low incidence but severe consequences are also notable. Some observational studies showed that treatment with GLP-1 RAs was associated with an increased OR value (2.24–29.4) of pancreatitis (12–14). However, the differences in gastrointestinal toxicities among different GLP-1 RAs are still not known. These problems pose concerns for physician about how to choose the safest and most appropriate medication therapy. In addition, there have been no recent systematic review updates, and further research on the seriousness and time-to-onset of various GLP-1-related gastrointestinal AEs is needed. Thus, this study aimed to characterize and compare gastrointestinal AEs of different marketed GLP-1RAs based on real-world data to provide evidence for physicians on the safety profiles of various GLP-1 RAs and how to choose the most appropriate GLP-1 RA medication.

## Methods

### Data sources

This retrospective pharmacovigilance study was conducted based on the FDA Adverse Event Reporting System (FAERS) database. FAERS is a publicly available post-marketing safety surveillance database that contains adverse event reports, medication error reports, and product quality complaints reported by health professionals, pharmaceutical manufacturers, lawyers, and individual patients (15, 16). Although FAERS is a US database, it receives AEs reports from all over the world. Therefore, the large size and global coverage of this open database make it particularly suitable for the analysis of spontaneous data reporting (15). FAERS database includes the following eight types of files: demographic and administrative information (DEMO), drug information (DRUG), adverse events (REAC), patient outcomes (OUTC), report sources (RPSR), start and end dates for reported drugs (THER), indications for use (INDI), and invalid reports (DELETED). All files recorded “primaryid” and “caseid” variables; therefore, the information about patients and AEs could be obtained by linking these variables in all files. DRUG and THER files recorded “drug_seq” variables; therefore, information about drug use and therapy could be obtained by linking “drug_seq” variables in these two files. All files are available at the FDA website (https://fis.fda.gov/extensions/FPDQDE-FAERS/FPD-QDE-FAERS.html).

**Figure 1:**
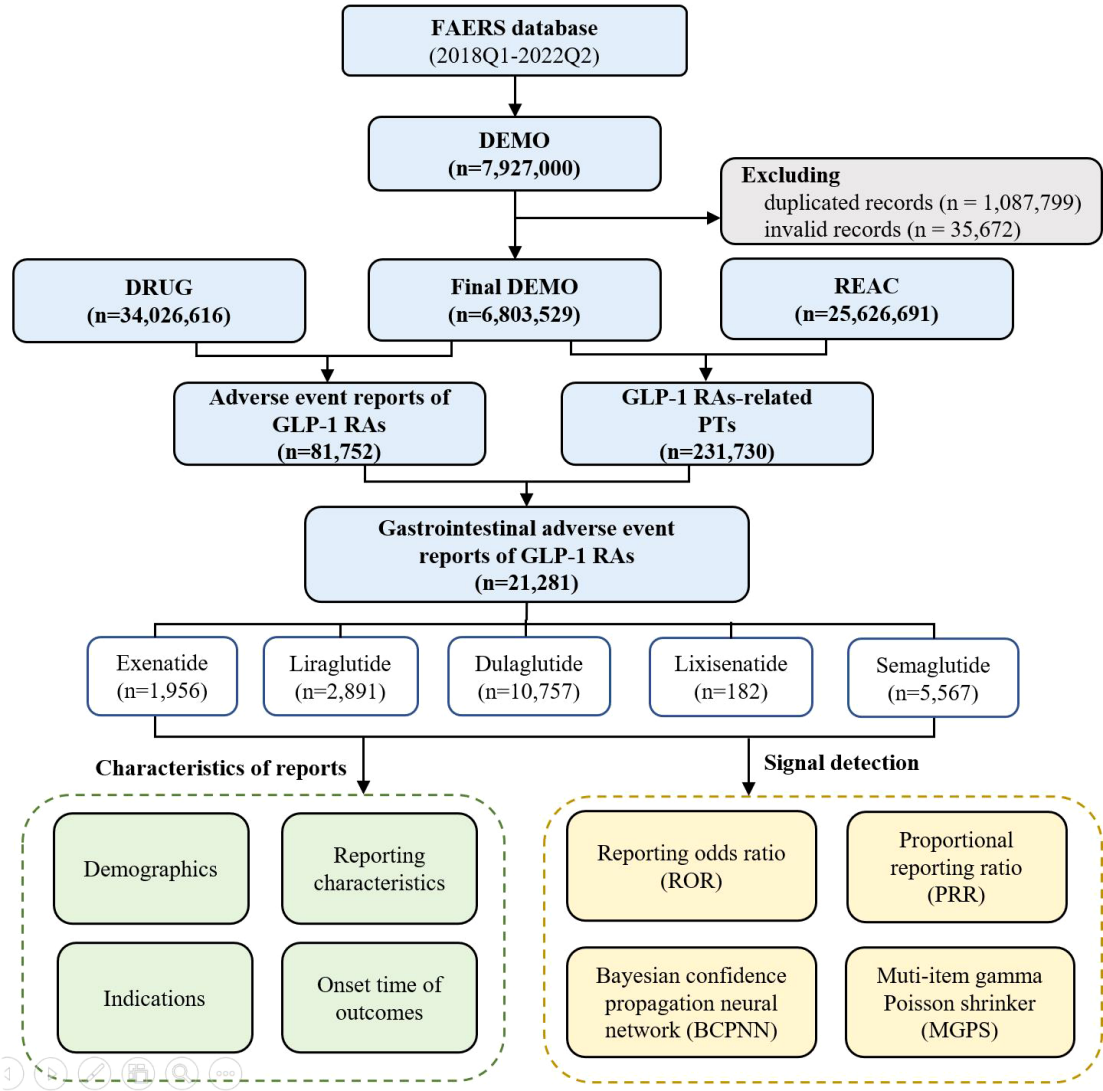
The flow diagram of selecting GLP-1 RA-associated AEs from FAERS database.

All reports between 1 January 2018 and 30 June 2022 were extracted for this analysis. We chose 2018 as the starting year because the most recent approved GLP-1 RA (semaglutide) except for tirzepatide was marketed in 2017, and the ending date was set as the most recent quarter when data were available. The study period was also the data analysis period, given that our research was a cross-sectional study. During the study period, a total of 7,927,000 reports were retrieved from the FAERS database. According to FDA’s recommendations, a two-step deduplication process was used to ensure the unique report: 1) selecting the higher “primaryid” when the “caseid” and “fda_dt” were the same, 2) selecting the latest “fda_dt” when “caseid” were the same, where “primaryid” was an unique number for identifying a AE report, “caseid” was an unique number for identifying a case, and “fda_dt” was the date when FDA received a case (17). Then, the expurgated data from the DELETED file was downloaded from FAERS to delete the invalid reports, finally reducing the number of reports to 6,803,529 (**Figure 1**).

FAERS is a publicly available and anonymous database. Therefore, approval and written informed consent were waived by the ethics review board of the Ethical Committee of West China Second University Hospital of Sichuan University.

### Data extraction

The generic and brand names of GLP-1 RAs approved by the FDA were used to identify adverse events of GLP-1 RAs in the DRUG files, including exenatide (BYETTA, BYDUREON), liraglutide (VICTOZA, SAXENDA), dulaglutide (TRULICITY), lixisenatide (ADLYXIN, SOLIQUA), and semaglutide (OZEMPIC, RYBELSUS, WEGOVY). Of note, albiglutide (TANZEUM) and tirzepatide (MOUNJARO) were not included in our study, given that albiglutide has been discontinued in the United States market since 26 July 2017, and tirzepatide was approved by the FDA in May 2022. The data for tirzepatide were too immature to support the analysis. The summary of approval dates for these GLP-1RAs is listed in **Supplementary Table 1**.

The AEs in REAC files are encoded by the preferred terms (PTs) in the Medical Dictionary for Regulatory Activities (MedDRA) terminology, which is comprised of 27 system organ classes (SOCs) (16). The structural hierarchy of MedDRA terminology has five levels: SOC (system organ class), HLGT (high level group term), HLT (high level term), PT (preferred term), and LLT (lowest level term) (17). Accordingly, the latest version of MedDRA 25.0 was used to classify AEs in reports at the relevant SOC level. In our study, all GLP-1 RA-induced PTs were analyzed, and we found that gastrointestinal AEs were most frequently reported in GLP-1 RAs. Therefore, the PTs of GLP-1 RAs below the SOC for gastrointestinal system disorders (SOC: 10017947) were analyzed. Gastrointestinal-related AEs were defined by 905 PTs, including nausea, vomiting, etc. The role codes for AEs had been assigned by reporters, including primary suspected (PS), secondary suspected drug (SS), concomitant (C), and interacting (I). To guarantee GLP-1 RAs were most likely to cause AEs during drug use, analysis reports were limited to those in which the “role_cod” of the drug was “PS” (primary suspected) in the DRUG files (18).

Demographics (gender, age), reporting characteristics (reporting region, year, occupation of reporters), and indications of reports of GLP-1 RA-associated gastrointestinal toxicities were analyzed. In addition, we calculated the time-to-onset of gastrointestinal AEs and the proportion of AE outcomes that were caused by different GLP-1 RAs. The calculation method of time-to-onset is the interval between the start time of GLP-1 RAs’ utilization and the time of AE occurrence (19). Reports with date errors (drug use time later than event occurrence time) and missing date data were excluded. Severe outcomes included death (grade 5), life-threatening conditions (grade 4), and outcomes causing hospitalization, disability, or a congenital anomaly (grade 3) (20). The proportion of reports with severe outcomes was calculated by dividing the number of reports with severe outcomes by the total number of reports.

### Data mining and statistical analysis

All characteristics and outcome measures of AE reports regarding GLP-1 RAs were evaluated descriptively. Categorical variables were reported as frequencies and percentages, and continuous variables were summarized as means and standard deviations or medians with interquartile ranges depending on the data distribution.

A disproportionality analysis model was performed to detect the potential signals of gastrointestinal AEs caused by GLP-1 RAs at both the class level and the drug generic name level (21). When a target drug is more likely to induce a target AE than all other drugs, it will typically get a higher score due to higher disproportionality (e.g., when semaglutide causes more gastrointestinal AEs than other drugs, it will get a higher disproportionality score) (21). Both frequentist methods (reporting odds ratio [ROR] (22) and proportional reporting ratio [PRR] (23)) and Bayesian methods (information component [IC] (24) and empirical Bayes geometric mean [EBGM] (25)) of disproportionality analysis were applied to investigate the association between a gastrointestinal AE and GLP-1 RAs. The equations and corresponding criteria of the four disproportionality algorithms are listed in **Supplementary Table 2**.

To improve the reliability of signals and avoid occurring false positive signals, a signal was regarded as positive only when it met all criteria of four algorithms simultaneously (26). Then, the signal strength of the top six most frequently reported gastrointestinal AEs of overall GLP-1 RAs in the FAERS database was ranked among different GLP-A RAs, and the top six AEs are nausea, vomiting, diarrhea, upper abdominal pain, constipation, and pancreatitis, respectively. A Kruskal–Wallis test was performed to compare the time-to-onset of gastrointestinal outcomes in different GLP-1 RAs. The severe proportion of gastrointestinal events in different GLP-1 RAs was compared using Fisher’s exact test or Pearson’s chi-squared test. Two-sided P-values of <0.05 were considered statistically significant. Data extraction was conducted by MySQL 8.0, and statistical analyses were performed using R 4.10.

## Results

### Descriptive analysis

From January 2018 to June 2022, a total of 231,730 GLP-1 RA-associated AEs reports were recorded, of which 21,281 reports of gastrointestinal AEs were identified. Of these, 1,956 gastrointestinal reports were for exenatide (9.19%), 2,891 for liraglutide (13.58%), 10,757 for dulaglutide (50.55%), 182 for lixisenatide (0.86%), and 5,567 (26.16%) for semaglutide (**Figure 1**).

The characteristics of AE reports for different GLP-1 RAs are presented in **Table 1**. More male patients reported gastrointestinal AEs from GLP-1 RAs (54.19%). The median age (interquartile range) of patients was 62 (54–70) years. The region with the highest number of reports was North America (91.40%), followed by Europe (4.10%), and Asia (2.55%). The number of gastrointestinal AEs steadily increased from 4,163 in 2018 to 5,048 in 2021, which reflected the increasingly clinical application of GLP-1 RAs. These AEs were mainly reported by consumers (74.28%) and physicians (11.04%). Gastrointestinal AEs were most frequently reported for unknown indications (50.37%) and diabetes mellitus (41.07%). The median time-to-onset of gastrointestinal AEs was 1 (IQR 0–24) days, and 77.44% of the AEs occurred within 30 days after excluding invalid reports. Outcomes causing hospitalization, disability, or a congenital anomaly (2,933, 13.78%) account for the most frequent outcomes in all cases.

**Table 1:** The characteristics of GLP-1 RAs-associated gastrointestinal reports.

Characteristics	All GLP-1 RAs N = 21,281	Exenatide N = 1,956	Liraglutide N = 2,819	Dulaglutide N = 10,757	Lixisenatide N = 182	Semaglutide N = 5,567
**Demographics**						
**Gender, n (%)**						
Female	7,384 (34.70%)	682 (34.87%)	891 (31.61%)	3,547 (32.97%)	54 (29.67%)	2,210 (39.70%)
Male	11,532 (54.19%)	1,209 (61.81%)	1,884 (66.83%)	5,172 (48.08%)	95 (52.20%)	3,172 (56.98%)
Unknown	2,365 (11.11%)	65 (3.32%)	44 (1.56%)	2,038 (18.95%)	33 (18.13%)	185 (3.32%)
**Age, n (%)**						
0–18	15 (0.07%)	1 (0.05%)	6 (0.21%)	5 (0.05%)	0 (0.00%)	3 (0.05%)
18–44	1,031 (4.84%)	74 (3.78%)	286 (10.15%)	329 (3.06%)	11 (6.04%)	331 (5.95%)
45–65	4,913 (23.09%)	533 (27.25%)	978 (34.69%)	1,772 (16.47%)	50 (27.47%)	1,580 (28.38%)
65+	4,446 (20.89%)	544 (27.81%)	712 (25.26%)	1,585 (14.73%)	46 (25.27%)	1,559 (28.00%)
Unknown	10,876 (51.11%)	804 (41.10%)	837 (29.69%)	7,066 (65.69%)	75 (41.21%)	2,094 (37.61%)
Median (IQR)	62 (54–70)	64 (56–70)	60 (50–68)	62 (54–70)	63 (53–70)	63 (54–71)
**Reporting characteristics**						
**Reporting region, n (%)**						
North America	19,450 (91.40%)	1,834 (93.76%)	2,331 (82.69%)	10,041 (93.34%)	149 (81.87%)	5,095 (91.52%)
Europe	872 (4.10%)	68 (3.48%)	148 (5.25%)	240 (2.23%)	13 (7.14%)	73 (1.31%)
Asia	542 (2.55%)	35 (1.79%)	163 (5.78%)	367 (3.41%)	6 (3.30%)	301 (5.41%)
Oceania	233 (1.09%)	18 (0.92%)	17 (0.60%)	32 (0.30%)	0 (0.00%)	29 (0.52%)
South America	96 (0.45%)	1 (0.05%)	119 (4.22%)	49 (0.46%)	8 (4.40%)	56 (1.01%)
Africa	9 (0.04%)	0 (0.00%)	2 (0.07%)	6 (0.06%)	0 (0.00%)	1 (0.02%)
Unknown	79 (0.37%)	0 (0.00%)	39 (1.38%)	22 (0.20%)	6 (3.30%)	12 (0.22%)
**Reporting year, n (%)**						
2018	4,163 (19.56%)	608 (31.08%)	1,128 (40.01%)	1,925 (17.90%)	57 (31.32%)	445 (7.99%)
2019	4,468 (21.00%)	624 (31.90%)	561 (19.90%)	2,547 (23.68%)	56 (30.77%)	680 (12.21%)
2020	4,642 (21.81%)	412 (21.06%)	480 (17.03%)	2,300 (21.38%)	27 (14.84%)	1,423 (25.56%)
2021	5,048 (23.72%)	236 (12.07%)	362 (12.84%)	2,696 (25.06%)	30 (16.48%)	1,724 (30.97%)
2022Q1–Q2*	2,952 (13.87%)	76 (3.89%)	283 (10.04%)	1,287 (11.96%)	12 (6.59%)	1,294 (23.24%)
Unknown	8 (0.04%)	0 (0.00%)	5 (0.18%)	2 (0.02%)	0 (0.00%)	1 (0.02%)
**Occupation of reporters, n (%)**						
Physician	2,349 (11.04%)	200 (10.22%)	422 (14.97%)	689 (6.41%)	55 (30.22%)	983 (17.66%)
Pharmacist	896 (4.21%)	26 (1.33%)	147 (5.21%)	205 (1.91%)	1 (0.55%)	517 (9.29%)
Health professional	1,263 (5.93%)	29 (1.48%)	159 (5.64%)	563 (5.23%)	16 (8.79%)	496 (8.91%)
Other health-professional	552 (2.59%)	22 (1.12%)	201 (7.13%)	137 (1.27%)	14 (7.69%)	178 (3.20%)
Consumer	15,807 (74.28%)	1,320 (67.48%)	1,871 (66.37%)	9,148 (85.04%)	96 (52.75%)	3,372 (60.57%)
Lawyer	14 (0.07%)	0 (0.00%)	11 (0.39%)	2 (0.02%)	0 (0.00%)	1 (0.02%)
Unknown	400 (1.88%)	359 (18.35%)	8 (0.28%)	13 (0.12%)	0 (0.00%)	20 (0.36%)
**Indications, n (%)**						
Diabetes mellitus	8,741 (41.07%)	1,154 (59.00%)	1,202 (42.64%)	3,926 (36.50%)	105 (57.69%)	2,354 (42.28%)
Others	1,822 (8.56%)	358 (18.30%)	563 (19.97%)	357 (3.32%)	8 (4.40%)	536 (9.63%)
Unknown	10,718 (50.36%)	444 (22.70%)	1,054 (37.39%)	6,474 (60.18%)	69 (37.91%)	2,677 (48.09%)
**Outcomes, n (%)**						
Death (Grade 5)	160 (0.75%)	24 (1.23%)	30 (1.06%)	69 (0.64%)	4 (2.20%)	33 (0.59%)
Life-Threatening (Grade 4)	191 (0.90%)	21 (1.07%)	45 (1.60%)	77 (0.72%)	0 (0.00%)	48 (0.86%)
Those causing hospitalization, disability, or congenital anomaly (Grade 3)	2,933 (13.78%)	239 (12.22%)	582 (20.65%)	1,176 (10.93%)	35 (19.23%)	901 (16.18%)
Required Intervention to Prevent (Grade 2)	160 (0.75%)	24 (1.23%)	30 (1.06%)	69 (0.64%)	4 (2.20%)	33 (0.59%)
Other Medical Event (Grade 1)	191 (0.90%)	21 (1.07%)	45 (1.60%)	77 (0.72%)	0 (0.00%)	48 (0.86%)
**The time to onset (days)**						
[0,30)	3,234 (15.20%)	228 (11.66%)	448 (0.00%)	1,381 (12.84%)	32 (17.58%)	1,145 (20.57%)
[30,60)	299 (1.41%)	18 (0.92%)	49 (0.00%)	58 (0.54%)	1 (0.55%)	173 (3.11%)
[60,90)	180 (0.85%)	11 (0.56%)	20 (0.00%)	41 (0.38%)	1 (0.55%)	107 (1.92%)
[90,120)	102 (0.48%)	4 (0.20%)	14 (0.00%)	20 (0.19%)	0 (0.00%)	64 (1.15%)
[120,150)	54 (0.25%)	1 (0.05%)	11 (0.00%)	14 (0.13%)	1 (0.55%)	27 (0.49%)
[150,180)	54 (0.25%)	4 (0.20%)	5 (0.00%)	13 (0.12%)	1 (0.55%)	31 (0.56%)
[180,360)	123 (0.58%)	5 (0.26%)	29 (0.00%)	32 (0.30%)	1 (0.55%)	56 (1.01%)
360+	130 (0.61%)	11 (0.56%)	46 (0.00%)	44 (0.41%)	0 (0.00%)	29 (0.52%)
Median (IQR)	1 (0–24)	1 (0–19.5)	3 (0–37.75)	1 (0–7)	0 (0–11)	4 (0–40)

*The first and second quarters of 2022. N, number of total gastrointestinal adverse event reports.

### AE signals associated with different GLP-1 RAs

**Figure 2:**
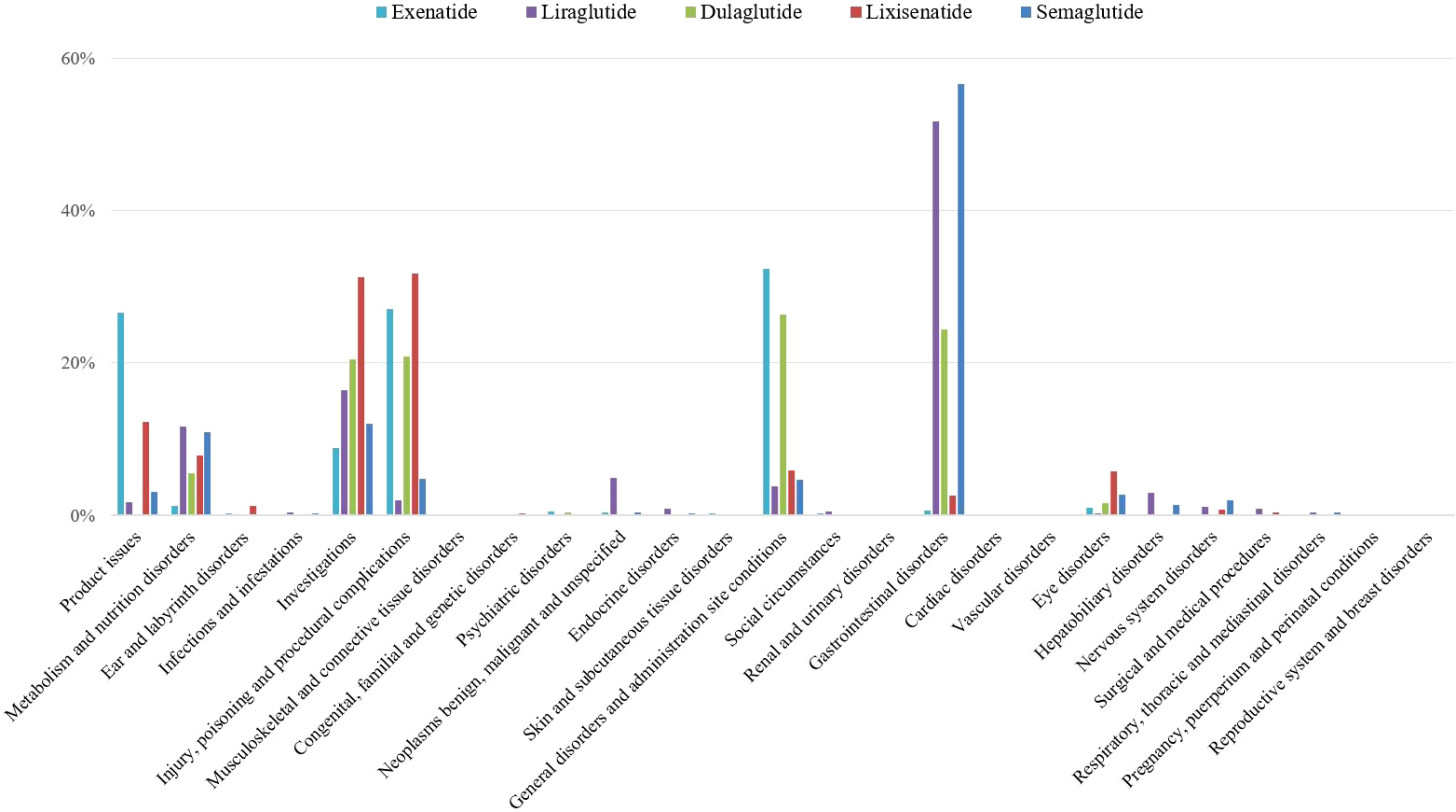
Proportion of positive signal for different GLP-1 RAs in system organ level.

All adverse events signals of GLP-1 RAs were detected by using four algorithms and their corresponding criteria. A total of 619 positive signal for GLP-1 RAs were observed, and the most AEs of positive signal were distributed in gastrointestinal system (**Figure 2**).

Within the SOC level, gastrointestinal system disorders in overall GLP-1 RAs were overreported compared with the background frequency (ROR 1.46, PRR 1.33, IC 0.41, EBGM 1.33), which were further attributed to liraglutide (ROR 2.39, PRR 1.84, IC 0.88, EBGM 1.33), dulaglutide (ROR 1.39, PRR 1.28, IC 0.36, EBGM 1.25), and semaglutide (ROR 3.00, PRR 2.10, IC 1.07, EBGM 2.10), while exenatide (ROR 0.57, PRR 0.63, IC −0.68, EBGM 0.63) and lixisenatide (ROR 0.51, PRR 0.57, IC −0.81, EBGM 0.57) did not show the association with gastrointestinal AEs (**Table 2**).

**Table 2:** Signal detection for GLP-1 RAs-associated gastrointestinal adverse events.

GLP-1 RA	The report number	ROR (95% CI)	PRR (χ^2^)	IC (IC025)	EBGM (EBGM05)
All GLP-1 RAs	2,1281	1.46 (1.44 to 1.49)	1.33 (2212.74)	0.41 (0.38)	1.33 (1.31)
Exenatide	1,956	0.57 (0.54 to 0.60)	0.63 (557.18)	−0.68 (-0.75)	0.63 (0.60)
Liraglutide	2,819	2.39 (2.28 to 2.51)	1.84 (1383.55)	0.88 (0.81)	1.84 (1.76)
Dulagutide	10,757	1.39 (1.36 to 1.42)	1.28 (843.37)	0.36 (0.32)	1.28 (1.25)
Lixisenatide	182	0.51 (0.44 to 0.60)	0.57 (74.96)	−0.81 (−1.04)	0.57 (0.49)
Semaglutide	5,567	3.00 (2.89 to 3.11)	2.10 (4082.54)	1.07 (1.02)	2.10 (2.03)

PRR, the proportional reporting ratio; ROR, the reporting odds ratio; IC, the information component; EBGM, the empirical Bayes geometric mean; CI, confidence interval; 95% CI, two‐sided for ROR, χ2, chi-squared; IC025 and EBGM05 lower one‐sided for IC and EBGM.

Furthermore, the positive signals of gastrointestinal AEs were classified as PT level (**Supplementary Table 3**). The top six most common gastrointestinal reports were nausea (8,988, 42.23%), diarrhea (4,666, 21.93%), vomiting (4,660, 21.90%), upper abdominal pain (2,082, 9.78%), constipation (1,790, 8.41%), and pancreatitis (1,752, 8.23%). All gastrointestinal AEs in the label of GLP-1 RAs were found in our study, which confirmed the reliability of this study. However, there were five disproportional signals that were not covered on the label that were detected for liraglutide (gastroesophageal reflux disease, gastritis, bezoar, breath odor, and intra-abdominal hematoma), one for dulaglutide (duodenogastric reflux), and three for semaglutide (breath odor, pancreatic failure, and mesenteric panniculitis).

Simultaneously, the signals of the top six common gastrointestinal AEs (nausea, diarrhea, vomiting, upper abdominal pain, constipation, and pancreatitis) for different GLP-1 RAs were detected and compared (**Table 3**). The results showed that semaglutide had the greatest risk of nausea (ROR, 7.41; 95% CI, 7.10–7.74), diarrhea (ROR, 3.55; 95% CI, 3.35–3.77), vomiting (ROR, 6.67; 95% CI, 6.32–7.05), and constipation (ROR, 6.17; 95% CI, 5.72–6.66); liraglutide had the greatest risk of upper abdominal pain (ROR, 4.63; 95% CI, 4.12–5.21). In addition, it was observed that use of any GLP-1 RA was associated with the risk of pancreatitis, and the signal strength of pancreatitis was ranked as follows: liraglutide (ROR, 32.67; 95% CI, 29.44–36.25) > semaglutide (ROR, 19.10, 95% CI, 17.26–21.13) > dulaglutide (ROR, 12.63; 95% CI, 11.76–13.56) > lixisenatide (ROR, 6.78; 95% CI 4.26–10.80) > exenatide (ROR, 4.91; 95% CI, 4.12–5.85).

**Table 3:** The difference of signal strength in the top six most frequently reported gastrointestinal adverse events among different GLP-1 RAs.

Preferred term (PT)	Drug	The report number	ROR	PRR	IC	EBGM
			(95% CI)	(χ^2^)	(IC025)	(EBGM05)
**Nausea**						
	Semaglutide	2,568	7.41 (7.10–7.74)	6.09 (11,184.65)	0.68 (0.57)	6.03 (5.78)
	Liraglutide	990	4.53 (4.24–4.85)	4.04 (2,339.60)	2.01 (1.90)	4.03 (3.77)
	Dulaglutide	4,566	3.75 (3.64–3.87)	3.43 (7,989.31)	1.76 (1.71)	3.38 (3.28)
	Exenatide	803	1.64 (1.52–1.76)	1.60 (187.63)	0.25 (-0.14)	1.60 (1.49)
	Lixisenatide	61	1.20 (0.93–1.55)	1.19 (1.92)	2.59 (2.52)	1.19 (0.92)
**Vomiting**						
	Semaglutide	1,462	6.67 (6.32–7.05)	6.01 (6,154.33)	0.45 (0.31)	5.95 (5.63)
	Liraglutide	576	4.35 (3.99–4.73)	4.08 (1,359.07)	2.02 (1.89)	4.06 (3.73)
	Dulaglutide	2,198	2.96 (2.84–3.09)	2.85 (2,654.91)	1.50 (1.43)	2.82 (2.70)
	Exenatide	397	1.38 (1.25–1.53)	1.37 (40.71)	−0.13 (−0.68)	1.37 (1.24)
	Lixisenatide	27	0.91 (0.62–1.33)	0.91 (0.23)	2.57 (2.48)	0.91 (0.62)
**Diarrhoea**						
	Semaglutide	1,266	3.55 (3.35–3.77)	3.29 (2,074.40)	−0.35 (−0.51)	3.28 (3.09)
	Liraglutide	472	2.20 (2.01–2.42)	2.12 (288.50)	1.08 (0.94)	2.12 (1.93)
	Dulaglutide	2,538	2.16 (2.08–2.25)	2.09 (1,466.71)	1.05 (0.99)	2.07 (1.99)
	Exenatide	357	0.78 (0.70–0.87)	0.78 (21.83)	−0.49 (−0.99)	0.78 (0.71)
	Lixisenatide	33	0.70 (0.50–0.99)	0.71 (4.03)	1.71 (1.62)	0.71 (0.50)
**Abdominal pain upper**						
	Liraglutide	291	4.63 (4.12–5.21)	4.49 (791.96)	0.23 (−0.00)	4.47 (3.97)
	Semaglutide	519	4.79 (4.38–5.23)	4.63 (1,477.81)	2.16 (1.97)	4.60 (4.21)
	Dulaglutide	1,097	3.16 (2.98–3.36)	3.10 (1,551.36)	1.62 (1.52)	3.07 (2.89)
	Lixisenatide	19	1.40 (0.89–2.21)	1.40 (2.18)	0.48 (−0.20)	1.40 (0.89)
	Exenatide	156	1.17 (1.00–1.38)	1.17 (4.01)	2.20 (2.06)	1.17 (1.00)
**Constipation**						
	Semaglutide	721	6.17 (5.72–6.66)	5.87 (2,913.63)	0.06 (−0.18)	5.82 (5.40)
	Liraglutide	247	3.55 (3.13–4.04)	3.47 (436.24)	1.79 (1.59)	3.46 (3.04)
	Dulaglutide	664	1.71 (1.58–1.85)	1.70 (190.80)	0.76 (0.64)	1.69 (1.57)
	Exenatide	152	1.04 (0.89–1.22)	1.04 (0.25)	−1.31 (−2.28)	1.04 (0.89)
	Lixisenatide	6	0.40 (0.18–0.89)	0.40 (5.38)	2.54 (2.42)	0.40 (0.18)
**Pancreatitis**						
	Liraglutide	386	32.67 (29.44–36.25)	30.96 (10,857.56)	2.27 (1.97)	30.02 (27.05)
	Semaglutide	400	19.10 (17.26–21.13)	18.52 (6,422.22)	4.91 (4.65)	17.94 (16.21)
	Dulaglutide	821	12.63 (11.76–13.56)	12.39 (8,029.15)	3.54 (3.42)	11.62 (10.82)
	Lixisenatide	18	6.78 (4.26–10.80)	6.71 (87.57)	2.75 (1.70)	6.71 (4.21)
	Exenatide	127	4.91 (4.12-5.85)	4.87 (387.52)	4.17 (3.96)	4.83 (4.05)

PRR, the proportional reporting ratio; ROR, the reporting odds ratio; IC, the information component; EBGM, the empirical Bayes geometric mean; CI, confidence interval; 95% CI, two‐sided for ROR, χ2, chi-squared; IC025 and EBGM05 lower one‐sided for IC and EBGM.

### Time-to-onset and severe proportion

The time-to-onset of gastrointestinal system disorders for each GLP-1 RA regimen is shown in **Table 4**. The result showed the time-to-onset of GLP-1 RAs had a statistical difference (p <0.001, Kruskal–Wallis **χ^2^** = 109.90). The longest median time-to-onset was 4 (IQR 0–40) days for semaglutide, while the shortest was 0 (IQR 0–11) day for lixisenatide, 1 (IQR 0–19.75) day for exenatide, 1 (IQR 0–7) day for dulaglutide, and 3 (IQR 0–36.75) days for liraglutide, respectively.

**Table 4:** Time-to-onset of GLP-1 RA-associated gastrointestinal AEs.

Interquartile range (IQR/day)	OverallGLP-1 RAs	Exenatide	Liraglutide	Dulaglutide	Lixisenatide	Semaglutide
First quartile	0	0	0	0	0	0
Median	1	1	3	1	0	4
Third quartile	24	19.75	36.75	7	11	40

**Figure 3:**
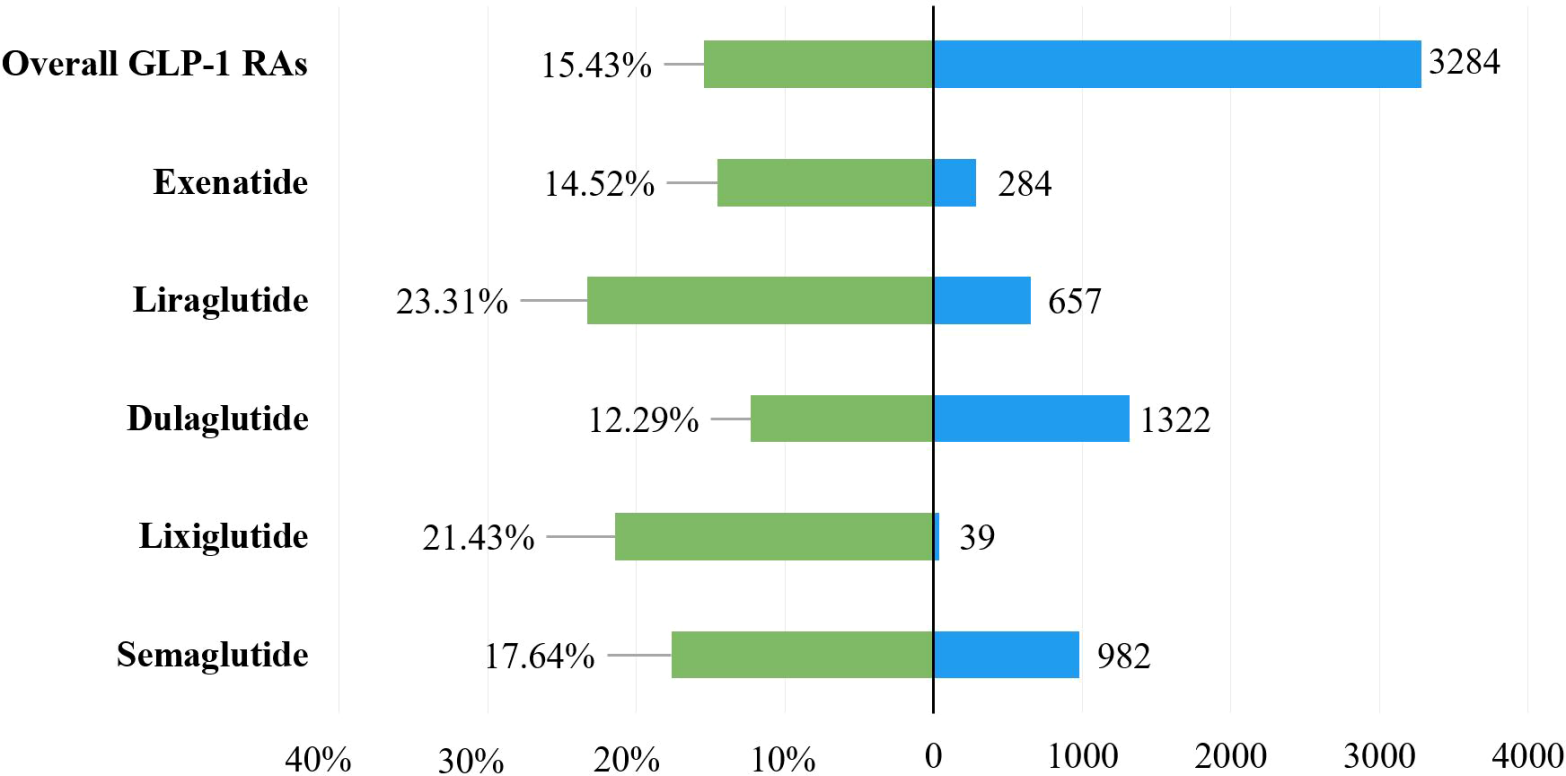
The case severe rate for GLP-1 RA-related gastrointestinal AEs.

To explore the prognosis of patients with gastrointestinal AEs after using GLP-1 RAs, this study evaluated the outcome of reports by severe proportions of outcomes. The corresponding results were presented in **Table 1** and **Figure 3**, and they showed a statistical difference in the severe proportions of GLP-1 RA-related gastrointestinal AEs among different GLP-1 RAs (p <0.001, Pearson’s **χ^2^** = 11,227). In all GLP-1 RAs, liraglutide (23.31%, 657 severe outcomes out of 2,819 cases) had the highest severe proportion of GLP-1 RA-associated gastrointestinal AEs, and the lowest for dulaglutide (12.29%, 1,322 severe outcomes out of 9,435 cases).

## Discussion

In this large real-world study, we found that GLP-1RAs are associated with an increased risk of gastrointestinal AEs, which were mainly attributed to liraglutide, dulaglutide, and semaglutide. The most frequently reported GLP-1 RA-induced gastrointestinal AE was nausea (8,988, 42.23%), diarrhea (4,666, 21.93%), and vomiting (4,660, 21.90%) in FAERS. This is consistent with prior studies, and a system review reported that ADRs of GLP-1 RAs tend to arise from the gastrointestinal system, with nausea, diarrhea, and vomiting occurring in up to 51%, 20%, and 19% of patients, respectively (27). These gastrointestinal AEs are related to the inhibition effect of GLP1-RAs on delay in gastric emptying and stimulation of neural circuitry (9, 28, 29).

In our study, semaglutide had the highest risk for nausea (ROR, 7.41; 95% CI, 7.10–7.74), vomiting (ROR, 6.67; 95% CI, 6.32–7.05), diarrhea (ROR, 3.55; 95% CI, 3.35–3.77), and constipation (ROR, 6.17; 95% CI, 5.72–6.66) when compared with background drugs. Semaglutide is a new generation of long-acting GLP-1RAs with a longer half-life than liraglutide after subcutaneous administration (183 *vs* 11–15 h) (30). Hence, the effects of gastrointestinal motility and neural circuitry of semaglutide may be more significant compared with those using other GLP-1 RAs. Our results were similar to those of previous studies, which reported that semagulutide had a higher risk of nausea, vomiting, diarrhea, and constipation compared with liraglutide (15).

Physician and patients have been expressed the concerns regarding a possible association of GLP-1 RAs treatment with pancreatitis. In our study, pancreatitis were the same disproportionality signals for all different GLP-1 RAs. These results were similar with prior studies. A cohort study revealed an increased risk of pancreatitis, and 36% of patients who used GLP-1 RAs or DPP- 4 inhibitors had elevated serum amylase or lipase (or both) levels compared with 18% of patients not taking these agents (31). Another case-control study showed that the use of incretin-mimetic therapies within 30 days (OR: 2.24, 95% CI: 1.36–3.68), or ranging from 30 days to 2 years was associated with an increased risk of acute pancreatitis (OR: 2.01, 95% CI: 1.37–3.18) compared with nonusers. Our study found that the signal strength of pancreatitis for different GLP-1 RA was ranked as following: liraglutide (ROR = 32.67) > semaglutide (ROR = 19.10) > dulaglutide (ROR = 12.63) > lixisenatide (ROR = 6.78) > exenatide (ROR = 4.91). It is suggested that patients being at risk of pancreatitis should not use any GLP-1 RAs, especially for liraglutide and semaglutide.

Our studies showed GLP-1 RA-associated gastrointestinal toxicities occurred 30 days (3,234, 77.44%) after the initiation of GLP-1RAs, and the median time-to-onset was 1 (IQR 0–24) days with a significant difference between different GLP-1 RA regimens. A systematic review was consistent with our results: gastrointestinal toxicities tended to occur early after treatment initiation with fluctuation over time, both in a type 2 diabetic population and a more general population (9). Time-to-onset analysis of gastrointestinal AEs may have been dependent upon the pharmacokinetic and pharmacodynamic properties of different GLP-1RAs. A “start low, go slow” strategy when initiating GLP-1 receptor agonists may reduce the likelihood of patients experiencing these events (32).

To further assess the severity of gastrointestinal AEs related to different GLP-1RAs, the severe proportion was compared among different GLP-1RA treatments. We observed that the severe rates had statistical significance in different GLP1- RAs. It was worth noting that liraglutide had the highest severe rate (23.31%, 657/2,819). Therefore, physicians should pay close attention to the health status of patients after AEs to avoid the progression to severe outcomes. LEADER trial showed that 32.2% patients experienced severe AEs in the liraglutide group for 4.5 years follow-up period, while these AE not only limited to gastrointestinal (33).

Although our study had significant advantages based on the FAERS database and data mining technology, there were also some limitations inherent to its observational design. First, the FAERS database is a self-reporting system with reporting randomness (e.g., existing selective, incomplete, inaccurate, untimely, and unverified reporting) and massive missing data (e.g., age and dosage). It is difficult to consider some confounders (such as dosage, duration of use, comorbidities, and drug combinations) that may influence the occurrence of gastrointestinal toxicity. Second, because the FAERS only contains cases with adverse events, the total number of patients receiving GLP-1RA treatment was low. Hence, the incidence rate of gastrointestinal AEs associated with GLP-1RA use cannot be estimated. Third, we focus only on gastrointestinal toxicity, and the deep relationship between GLP-1RAs and other system organ classes remains unknown. Further clinical trials or observational studies are needed to confirm our results. Finally, disproportionality analysis based on FAERS showed neither causality nor quantified risk due to the lack of pharmacological mechanism study but only showed an evaluation of the signal strength, which was just a statistical association (34). Like other pharmacovigilance studies, Frequentist and Bayesian are indexes of increased risk in AE reports, whether a causality exists and needs to be further validated by basic research.

## Conclusion

We explored the relationship between GLP-1 RAs and gastrointestinal AEs from various perspectives and quantified the potential risks based on a comprehensive and systematic retrospective analysis of the FAERS database. GLP-1 RAs were significantly associated with gastrointestinal AEs, and the association was further attributed to liraglutide, dulaglutide, and semaglutide. In addition, semaglutide had the greatest risk of nausea, diarrhea, vomiting, constipation, and pancreatitis, while liraglutide had the greatest risk when it came to upper abdominal pain. Furthermore, the time-to-onset and severe outcomes of GLP-1 RAs-induced gastrointestinal AEs were also discussed, which was helpful for clinical practice and drug monitoring to a certain extent. Our study provided valuable evidence for early clinical interventions and the identification of the risk of gastrointestinal toxicities.

## Data availability statement

The raw data supporting the conclusions of this article will be made available by the authors, without undue reservation.

## Ethics statement

Ethical approval was not provided for this study on human participants because FAERS (FDA Adverse Event Reporting System) is publicly available and anonymous database, so approval and written informed consent were waived by the ethics review board of the Ethical Committee of West China Second University Hospital of Sichuan University. Written informed consent from the participants’ legal guardian/next of kin was not required to participate in this study in accordance with the national legislation and the institutional requirements.

## Author contributions

LC and LL contributed to conception and study design, and took responsibility for the collection, integrity, and accuracy of the data. All authors drafted the manuscript, participated in data analyses and interpretation, and revisions of the manuscript and approved the final version.
