# Supplementary material for: Association between different GLP-1 receptor agonists and gastrointestinal adverse reactions: A real-world disproportionality study based on FDA adverse event reporting system database
Source: Front Endocrinol (Lausanne). 2022 Dec 7;13:1043789. doi: 10.3389/fendo.2022.1043789 (PMC9770009; doi:10.3389/fendo.2022.1043789)
Supplement: Supplementary file 1 [file Table_1.docx]

Supplementary Material

**Supplementary Table 1. Summary of FDA-approved GLP-1RAs**

| **Generic name** | **Brand name** | **Approval date** |
| --- | --- | --- |
| Exenatide | Bydureon, Bydureon Bcise, Byetta | Apr. 2005 |
| Liraglutide | Saxenda, Victoza | Jan. 2010 |
| Albiglutide | Eperzan, Tanzeum | Apr. 2014 |
| Dulaglutide | Trulicity | Sep. 2014 |
| Lixisenatide | Adlyxin, Lyxumia | Jul. 2016 |
| Semaglutide | Ozempic, Rybelsus | Dec. 2017 |
| Tirzepatide | Mounjaro | May 2022 |

**Supplementary Table 2. Four major algorithms used for signal detection.**

| Algorithms | Equation | Criteria |
| --- | --- | --- |
| ROR | ROR=ad/bc | lower limit of 95% CI>1, a≥3 |
|  | 95%CI=e^ln(ROR)±1.96(1/a+1/b+1/c+1/d)^0.5^ |  |
| PRR | PRR=a(c+d)/c/(a+b) | PRR≥2, χ^2^≥4, a≥3 |
|  | χ^2^=[(ad-bc)^2](a+b+c+d)/[(a+b)(c+d)(a+c)(b+d)] |  |
| BCPNN | IC=log_2_a(a+b+c+d)/(a+c)/(a+b) | IC025>0, a≥3 |
|  | 95%CI=E(IC) ± 2V(IC)^0.5 |  |
|  | r=(a+b+c+d)^2/(a+b+1)/(a+c+1) |  |
|  | E(IC)=log_2_a(a+b+c+d)^2/(a+b+c+d+r)/(a+b)/(a+c) |  |
|  | V(IC)=1/ln2(b+c+d+r-1)/(a+1)/(a+b+c+d+r+1)+(2+b+c+2d)/(a+b+1)/(a+b+c+d+r+3) |  |
|  | IC025=E(IC)- 2V(IC)^0.5 |  |
| MGPS | EBGM=a(a+b+c+d)/(a+c)/(a+b) | EBGM05≥2, a≥3 |
|  | 95%CI=e^ln(EBGM)±1.96(1/a+1/b+1/c+1/d)^0.5^ |  |
|  | EBGM05=e^ln(EBGM)-1.96(1/a+1/b+1/c+1/d)^0.5^ |  |

Equation: a, number of reports containing both the suspect drug and the suspect adverse drug reaction; b, number of reports containing the suspect adverse drug reaction with other medications (except the drug of interest); c, number of reports containing the suspect drug with other adverse drug reactions (except the event of interest); d, number of reports containing other medications and other adverse drug reactions. ROR, reporting odds ratio; CI, confidence interval; N, the number of co-occurrences; PRR, proportional reporting ratio; χ^2^, chi-squared; BCPNN, Bayesian confidence propagation neural network; IC, information component; IC025, the lower limit of the 95% one-sided CI of the IC; MGPS, multi-item gamma Poisson shrinker; EBGM, empirical Bayesian geometric mean; EBGM05, the lower 95% one-sided CI of EBGM.

**Table 3. Signal strength of gastrointestinal adverse events reports of GLP-1 RAs at the Preferred Term (PT) level in FAERS database**

| **Preferred term (PT)** | **The report number** | **ROR (95%CI)** | **PRR  (χ^2^)** | **IC (IC025)** | **EBGM (EBGM05)** |
| --- | --- | --- | --- | --- | --- |
| **Exenatide** |  |  |  |  |  |
| Pancreatitis | 127 | 4.91 (4.12-5.85) | 4.87 (387.52) | 2.27 (1.97) | 4.83 (4.05) |
| Eructation | 72 | 7.63 (6.04-9.64) | 7.60 (406.16) | 2.91 (2.44) | 7.49 (5.93) |
| Impaired gastric emptying | 47 | 9.03 (6.76-12.06) | 9.00 (328.17) | 3.15 (2.51) | 8.85 (6.63) |
| Pancreatic disorder | 15 | 4.84 (2.91-8.05) | 4.83 (45.16) | 2.26 (1.23) | 4.80 (2.88) |
| Abdominal mass | 11 | 7.00 (3.86-12.70) | 6.99 (55.67) | 2.79 (1.38) | 6.90 (3.81) |
| Stomach mass | 8 | 13.18 (6.53-26.63) | 13.18 (87.54) | 3.68 (1.51) | 12.84 (6.36) |
| Pancreatic cyst | 6 | 4.84 (2.16-10.82) | 4.84 (18.08) | 2.26 (0.54) | 4.80 (2.15) |
| Pancreatic mass | 5 | 7.12 (2.94-17.23) | 7.12 (25.91) | 2.81 (0.63) | 7.03 (2.91) |
| Diabetic gastroparesis | 3 | 21.37 (6.71-67.99) | 21.36 (55.66) | 4.36 (0.36) | 20.46 (6.43) |
| **Liraglutide** |  |  |  |  |  |
| Nausea | 990 | 4.53 (4.24-4.85) | 4.04 (2339.60) | 2.01 (1.90) | 4.03 (3.77) |
| Vomiting | 576 | 4.35 (3.99-4.73) | 4.08 (1359.07) | 2.02 (1.89) | 4.06 (3.73) |
| Pancreatitis | 386 | 32.67 (29.44-36.25) | 30.96 (10857.56) | 4.91 (4.65) | 30.02 (27.05) |
| Abdominal pain upper | 291 | 4.63 (4.12-5.21) | 4.49 (791.96) | 2.16 (1.97) | 4.47 (3.97) |
| Constipation | 247 | 3.55 (3.13-4.04) | 3.47 (436.24) | 1.79 (1.59) | 3.46 (3.04) |
| Abdominal discomfort | 220 | 3.66 (3.20-4.19) | 3.58 (410.55) | 1.84 (1.62) | 3.57 (3.12) |
| Abdominal pain | 212 | 3.04 (2.65-3.49) | 2.98 (281.33) | 1.57 (1.36) | 2.98 (2.60) |
| Eructation | 172 | 38.91 (33.35-45.40) | 38.00 (5961.73) | 5.19 (4.70) | 36.57 (31.35) |
| Dyspepsia | 158 | 5.70 (4.87-6.68) | 5.60 (595.82) | 2.48 (2.20) | 5.57 (4.76) |
| Abdominal distension | 154 | 4.99 (4.25-5.85) | 4.90 (477.76) | 2.29 (2.01) | 4.88 (4.16) |
| Gastrooesophageal reflux disease | 110 | 4.49 (3.72-5.42) | 4.44 (292.31) | 2.14 (1.82) | 4.42 (3.66) |
| Flatulence | 108 | 6.50 (5.37-7.86) | 6.42 (491.52) | 2.67 (2.32) | 6.38 (5.27) |
| Pancreatitis acute | 98 | 15.19 (12.42-18.57) | 14.99 (1261.23) | 3.89 (3.40) | 14.78 (12.09) |
| Gastritis | 34 | 4.52 (3.22-6.33) | 4.50 (92.19) | 2.16 (1.54) | 4.48 (3.20) |
| Retching | 30 | 4.97 (3.47-7.12) | 4.95 (94.23) | 2.30 (1.61) | 4.93 (3.44) |
| Impaired gastric emptying | 22 | 8.56 (5.62-13.03) | 8.53 (145.09) | 3.08 (2.07) | 8.47 (5.56) |
| Gastrointestinal sounds abnormal | 14 | 9.74 (5.75-16.50) | 9.73 (108.52) | 3.27 (1.87) | 9.64 (5.69) |
| Obstructive pancreatitis | 14 | 43.98 (25.72-75.20) | 43.90 (560.93) | 5.39 (2.74) | 42.00 (24.56) |
| Pancreatic cyst | 13 | 21.75 (12.54-37.71) | 21.71 (251.11) | 4.41 (2.34) | 21.25 (12.26) |
| Regurgitation | 12 | 14.14 (7.99-25.01) | 14.12 (144.11) | 3.80 (2.00) | 13.92 (7.87) |
| Pancreatic disorder | 10 | 6.58 (3.53-12.26) | 6.57 (46.92) | 2.71 (1.25) | 6.53 (3.51) |
| Pancreatitis chronic | 8 | 12.60 (6.27-25.32) | 12.58 (84.20) | 3.64 (1.49) | 12.43 (6.19) |
| Vomiting projectile | 8 | 10.18 (5.07-20.44) | 10.17 (65.44) | 3.33 (1.36) | 10.07 (5.02) |
| Breath odour | 6 | 9.82 (4.39-21.96) | 9.81 (47.01) | 3.28 (1.02) | 9.72 (4.35) |
| Pancreatic mass | 6 | 17.55 (7.82-39.37) | 17.54 (91.86) | 4.11 (1.28) | 17.24 (7.68) |
| Gastric dilatation | 4 | 7.32 (2.73-19.57) | 7.31 (21.63) | 2.86 (0.40) | 7.26 (2.72) |
| Pancreatitis necrotising | 4 | 7.64 (2.86-20.44) | 7.64 (22.89) | 2.92 (0.42) | 7.58 (2.83) |
| Bezoar | 3 | 6.88 (2.21-21.43) | 6.88 (14.96) | 2.77 (0.03) | 6.84 (2.20) |
| Intra-abdominal haematoma | 3 | 7.54 (2.42-23.48) | 7.54 (16.87) | 2.90 (0.07) | 7.48 (2.40) |
| Pancreatic pseudocyst | 3 | 17.54 (5.60-54.98) | 17.54 (45.93) | 4.11 (0.32) | 17.24 (5.50) |
| **Dulaglutide** |  |  |  |  |  |
| Nausea | 4566 | 3.75 (3.64-3.87) | 3.43 (7989.31) | 1.76 (1.71) | 3.38 (3.28) |
| Vomiting | 2198 | 2.96 (2.84-3.09) | 2.85 (2654.91) | 1.50 (1.43) | 2.82 (2.70) |
| Abdominal pain upper | 1097 | 3.16 (2.98-3.36) | 3.10 (1551.36) | 1.62 (1.52) | 3.07 (2.89) |
| Abdominal discomfort | 933 | 2.83 (2.65-3.02) | 2.78 (1057.40) | 1.46 (1.36) | 2.75 (2.58) |
| Pancreatitis | 821 | 12.63 (11.76-13.56) | 12.39 (8029.15) | 3.54 (3.42) | 11.62 (10.82) |
| Eructation | 618 | 28.03 (25.73-30.53) | 27.61 (13659.53) | 4.58 (4.41) | 23.92 (21.96) |
| Dyspepsia | 530 | 3.48 (3.20-3.80) | 3.45 (907.85) | 1.77 (1.63) | 3.40 (3.12) |
| Abdominal distension | 518 | 3.05 (2.79-3.33) | 3.02 (692.15) | 1.58 (1.45) | 2.99 (2.74) |
| Flatulence | 412 | 4.56 (4.13-5.03) | 4.52 (1102.76) | 2.15 (1.99) | 4.43 (4.01) |
| Impaired gastric emptying | 149 | 11.08 (9.39-13.08) | 11.05 (1279.38) | 3.38 (3.06) | 10.44 (8.84) |
| Pancreatitis acute | 102 | 2.83 (2.33-3.45) | 2.83 (118.76) | 1.49 (1.17) | 2.80 (2.30) |
| Pancreatic disorder | 72 | 8.97 (7.08-11.37) | 8.96 (483.74) | 3.10 (2.62) | 8.56 (6.75) |
| Vomiting projectile | 41 | 9.89 (7.22-13.55) | 9.88 (309.37) | 3.23 (2.52) | 9.39 (6.86) |
| Gastrointestinal sounds abnormal | 25 | 3.17 (2.14-4.71) | 3.17 (36.51) | 1.65 (0.97) | 3.13 (2.11) |
| Pancreatitis necrotising | 20 | 7.15 (4.57-11.19) | 7.15 (101.52) | 2.79 (1.80) | 6.90 (4.41) |
| Infrequent bowel movements | 16 | 4.13 (2.51-6.78) | 4.13 (37.03) | 2.02 (1.08) | 4.05 (2.47) |
| Pancreatitis chronic | 15 | 4.33 (2.59-7.22) | 4.32 (37.40) | 2.08 (1.10) | 4.24 (2.54) |
| Breath odour | 13 | 3.90 (2.25-6.76) | 3.90 (27.40) | 1.94 (0.90) | 3.83 (2.21) |
| Defaecation disorder | 9 | 4.54 (2.34-8.80) | 4.54 (24.21) | 2.15 (0.81) | 4.45 (2.30) |
| Obstructive pancreatitis | 8 | 4.46 (2.21-8.99) | 4.46 (20.90) | 2.13 (0.70) | 4.37 (2.16) |
| Pancreatic mass | 8 | 4.26 (2.11-8.60) | 4.26 (19.49) | 2.06 (0.66) | 4.18 (2.07) |
| Pancreatic enlargement | 4 | 7.62 (2.80-20.76) | 7.62 (22.04) | 2.88 (0.40) | 7.34 (2.70) |
| Diabetic gastroparesis | 3 | 7.92 (2.49-25.19) | 7.92 (17.33) | 2.93 (0.07) | 7.61 (2.39) |
| Duodenogastric reflux | 3 | 7.35 (2.31-23.35) | 7.35 (15.79) | 2.83 (0.04) | 7.09 (2.23) |
| **Lixisenatide** |  |  |  |  |  |
| Pancreatitis | 18 | 6.78 (4.26-10.80) | 6.71 (87.57) | 2.75 (1.70) | 6.71 (4.21) |
| Pancreatitis acute | 8 | 5.81 (2.90-11.65) | 5.79 (31.65) | 2.53 (0.95) | 5.78 (2.88) |
| Impaired gastric emptying | 4 | 7.40 (2.77-19.77) | 7.39 (22.06) | 2.88 (0.40) | 7.38 (2.76) |
| Pancreatic disorder | 3 | 9.42 (3.03-29.29) | 9.41 (22.50) | 3.23 (0.15) | 9.39 (3.02) |
| **Semaglutide** |  |  |  |  |  |
| Nausea | 2568 | 7.41 (7.10-7.74) | 6.09 (11184.65) | 2.59 (2.52) | 6.03 (5.78) |
| Vomiting | 1462 | 6.67 (6.32-7.05) | 6.01 (6154.33) | 2.57 (2.48) | 5.95 (5.63) |
| Diarrhoea | 1266 | 3.55 (3.35-3.77) | 3.29 (2074.40) | 1.71 (1.62) | 3.28 (3.09) |
| Constipation | 721 | 6.17 (5.72-6.66) | 5.87 (2913.63) | 2.54 (2.42) | 5.82 (5.40) |
| Abdominal pain upper | 519 | 4.79 (4.38-5.23) | 4.63 (1477.81) | 2.20 (2.06) | 4.60 (4.21) |
| Abdominal pain | 418 | 3.48 (3.16-3.84) | 3.40 (710.88) | 1.76 (1.61) | 3.39 (3.07) |
| Pancreatitis | 400 | 19.10 (17.26-21.13) | 18.52 (6422.22) | 4.17 (3.96) | 17.94 (16.21) |
| Abdominal distension | 360 | 6.82 (6.14-7.58) | 6.65 (1714.57) | 2.72 (2.54) | 6.58 (5.92) |
| Abdominal discomfort | 358 | 3.43 (3.09-3.82) | 3.36 (596.05) | 1.74 (1.58) | 3.35 (3.01) |
| Eructation | 339 | 46.15 (41.25-51.63) | 44.92 (13459.81) | 5.38 (5.05) | 41.58 (37.17) |
| Flatulence | 291 | 10.30 (9.16-11.58) | 10.08 (2342.21) | 3.31 (3.09) | 9.91 (8.82) |
| Dyspepsia | 266 | 5.55 (4.92-6.27) | 5.46 (962.25) | 2.44 (2.23) | 5.41 (4.79) |
| Gastrooesophageal reflux disease | 182 | 4.29 (3.70-4.97) | 4.24 (449.12) | 2.08 (1.83) | 4.22 (3.64) |
| Gastrointestinal disorder | 161 | 3.37 (2.89-3.94) | 3.34 (263.96) | 1.74 (1.49) | 3.33 (2.85) |
| Retching | 73 | 7.03 (5.58-8.87) | 7.00 (370.91) | 2.79 (2.34) | 6.92 (5.49) |
| Pancreatitis acute | 65 | 5.73 (4.48-7.32) | 5.70 (249.66) | 2.50 (2.04) | 5.65 (4.42) |
| Impaired gastric emptying | 50 | 11.35 (8.57-15.03) | 11.31 (460.55) | 3.47 (2.81) | 11.10 (8.38) |
| Gastritis | 41 | 3.14 (2.31-4.27) | 3.13 (59.16) | 1.64 (1.12) | 3.12 (2.29) |
| Gastrointestinal sounds abnormal | 33 | 13.43 (9.51-18.99) | 13.40 (369.72) | 3.71 (2.78) | 13.10 (9.27) |
| Vomiting projectile | 26 | 19.56 (13.22-28.93) | 19.52 (441.14) | 4.24 (2.95) | 18.88 (12.76) |
| Bowel movement irregularity | 22 | 4.62 (3.03-7.03) | 4.61 (61.69) | 2.20 (1.38) | 4.58 (3.01) |
| Obstructive pancreatitis | 16 | 29.15 (17.62-48.21) | 29.11 (412.34) | 4.79 (2.73) | 27.69 (16.74) |
| Regurgitation | 12 | 8.14 (4.60-14.40) | 8.14 (74.02) | 3.01 (1.58) | 8.03 (4.54) |
| Epigastric discomfort | 11 | 4.63 (2.55-8.37) | 4.62 (30.97) | 2.20 (0.99) | 4.59 (2.54) |
| Pancreatitis necrotising | 8 | 8.88 (4.42-17.86) | 8.88 (55.01) | 3.13 (1.27) | 8.75 (4.35) |
| Infrequent bowel movements | 7 | 5.67 (2.69-11.94) | 5.67 (26.64) | 2.49 (0.81) | 5.62 (2.67) |
| Breath odour | 6 | 5.66 (2.53-12.65) | 5.66 (22.76) | 2.49 (0.67) | 5.61 (2.51) |
| Pancreatic cyst | 6 | 5.71 (2.55-12.76) | 5.71 (23.05) | 2.50 (0.67) | 5.66 (2.53) |
| Pancreatic failure | 6 | 5.47 (2.45-12.23) | 5.47 (21.69) | 2.44 (0.64) | 5.42 (2.43) |
| Pancreatic mass | 6 | 10.11 (4.51-22.68) | 10.11 (48.35) | 3.31 (1.03) | 9.94 (4.43) |
| Pancreatitis chronic | 6 | 5.42 (2.43-12.12) | 5.42 (21.43) | 2.43 (0.63) | 5.38 (2.41) |
| Faecal vomiting | 4 | 12.20 (4.53-32.87) | 12.20 (40.22) | 3.58 (0.61) | 11.95 (4.44) |
| Pancreatitis relapsing | 4 | 10.87 (4.04-29.24) | 10.86 (35.12) | 3.42 (0.57) | 10.67 (3.97) |
| Mechanical ileus | 3 | 8.36 (2.67-26.14) | 8.35 (19.13) | 3.04 (0.11) | 8.24 (2.64) |
| Mesenteric panniculitis | 3 | 20.22 (6.39-64.02) | 20.22 (52.84) | 4.29 (0.35) | 19.53 (6.17) |
| Pancreatic enlargement | 3 | 18.00 (5.70-56.86) | 17.99 (46.61) | 4.13 (0.33) | 17.45 (5.52) |

PRR, the proportional reporting ratio; ROR, the reporting odds ratio; IC, the information component; EBGM, the empirical Bayes geometric mean; CI, confidence interval; 95% CI, two‐sided for ROR, χ2, chi-squared; IC025 and EBGM05 lower one‐sided for IC and EBGM.
